# Dyadic Psychopathology and Adjustment to Parenthood in Families With and Without Eating Disorder History—Findings From a Longitudinal Study

**DOI:** 10.1002/eat.24338

**Published:** 2024-11-27

**Authors:** Jana Katharina Throm, Annica Franziska Dörsam, Nadia Micali, Hubert Preissl, Katrin Elisabeth Giel

**Affiliations:** ^1^ Department of Psychosomatic Medicine and Psychotherapy University Hospital Tuebingen Tuebingen Germany; ^2^ Centre of Excellence for Eating Disorders Tuebingen (KOMET) University Hospital Tuebingen Tuebingen Germany; ^3^ Center for Eating and Feeding Disorders Research, Mental Health Center Ballerup Copenhagen University Hospital—Mental Health Services CPH Copenhagen Denmark; ^4^ Great Ormond Street Institute of Child Health University College London London UK; ^5^ Institute for Biological Psychiatry, Mental Health Centre Sct Hans Copenhagen University Hospital—Mental Health Services Copenhagen Denmark; ^6^ Institute for Diabetes Research and Metabolic Diseases (IDM) of the Helmholtz Center Munich at the University of Tuebingen, fMEG Center; German Centre for Diabetes Research (DZD) Tuebingen Baden‐Wuerttemberg Germany; ^7^ Department of Internal Medicine IV, Division of Endocrinology, Diabetology, and Nephrology University Hospital Tuebingen Tuebingen Baden‐Wuerttemberg Germany; ^8^ Institute of Pharmaceutical Sciences, Department of Pharmacy and Biochemistry Eberhard Karls University Tübingen Tübingen Germany; ^9^ German Center for Mental Health (DZPG) Tübingen Germany

**Keywords:** adjustment, children, eating disorder, fathers, mothers, parents, psychopathology

## Abstract

**Objective:**

Transition to parenthood is a vulnerable period for individual health and partnership quality. This study investigated parental health and partnership after childbirth in families with and without maternal eating disorder (ED) history. We report longitudinal data on parental ED psychopathology, depressive symptoms, and adjustment, including dyadic associations.

**Method:**

Data derived from the prospective multi‐method cohort study EMKIE. Women with (*n* = 24) and without (*n* = 33) ED history and their partners took part from late pregnancy to 10 months postpartum and completed the Eating Disorder Examination Questionnaire, the Patient Health Questionnaire, and the Maternal Adjustment and Maternal Attitudes Questionnaire or the paternal equivalent.

**Results:**

ED psychopathology increased in mothers in both groups. Mothers in the ED group had more severe ED psychopathology, higher depression scores, and lower levels of adjustment to motherhood compared to the control group across all measurement points. No group differences emerged between partners, but ED psychopathology increased in partners of women with ED history over time. A negative correlation between maternal ED severity and paternal adjustment was observed in the ED group.

**Discussion:**

After childbirth, mothers with ED history experienced mental health deterioration and adjustment difficulties and fathers struggled with paternal adjustment if their partner was affected by severe ED symptoms. These results emphasize the need for close monitoring and consistent care of women with ED during this vulnerable period and highlight adjustment needs of partners of severely affected women. Further qualitative approaches are needed to deepen the knowledge of paternal experiences during this period.

**Summary**

- In families with maternal ED history, transition to parenthood is characterized by an increased burden in terms of mothers experiencing mental health deterioration and adjustment difficulties and fathers struggling with paternal adjustment if their partner is affected by severe ED symptoms.
- The paternal role may also be supportive in the family system affected by ED history.
- Couples with ED history may need more support during the vulnerable period after childbirth.

## 1 Introduction

Transition to parenthood is a special time in couples' lives, associated with a decline in relationship functioning (Doss and Rhoades 2017), changes in parental eating behavior (e.g., skipping meals, irregular eating) (Versele et al. 2021) and risk of mental health deterioration (Smythe, Petersen, and Schartau 2022).

A history of eating disorders (ED) adds an additional burden and intensifies the typical challenges associated with the transition to parenthood (Sadeh‐Sharvit et al. 2020). ED psychopathology has been reported to increase during the postpartum period, especially in women with anorexia nervosa (AN) and bulimia nervosa (BN) and rates of postpartum depression are higher in women with ED than in the general population (Astrachan‐Fletcher et al. 2008; Makino, Yasushi, and Tsutsui 2020). Koubaa, Hällström, and Hirschberg (2008) found that mothers with a history of AN or BN before pregnancy had lower maternal adjustment 3 months after delivery than mothers without ED history. Furthermore, couples with ED history reported decreased relationship satisfaction (Dick et al. 2013), which may be exacerbated in the postpartum period. Qualitative studies with partners of individuals with ED provide consistent evidence of overwhelming feelings and psychosocial distress in general (Batchelor et al. 2022; Linville et al. 2016; O'Connor, Daly, and Higgins 2019; Schmit and Bell 2017). However, no study yet investigated the mental health outcomes in partners of women with ED history in the vulnerable period of transition to parenthood.

This study investigated parental mental health and dyadic partnership in families with and without maternal ED history by reporting longitudinal data on eating behavior, depressive symptoms, and adjustment to parenthood in mothers and their partners in the first year postpartum. We also investigated potential associations between parental psychopathology.

We hypothesized that

1. ED psychopathology and depressive symptoms would be more pronounced in women with ED history compared to women without ED history at all time points.
2. ED psychopathology and depressive symptoms would increase after childbirth in mothers with and without ED history.
3. Adjustment to parenthood would be lower in mothers with ED history compared to mothers without ED history.
4. Partners of women with ED history would experience more ED and depressive symptoms and more difficulties in adjusting to parenthood than partners of women without ED history in the first year after childbirth.

## 2 Method

This study was part of the family cohort study EMKIE, conducted at the Department of Psychosomatic Medicine and Psychotherapy at the University Hospital Tübingen. The EMKIE study assesses the influence of maternal ED on the family system from the third trimester of pregnancy until 42 months postpartum. More information on study design, recruitment process, and inclusion criteria has been described elsewhere (Doersam et al. 2022, 2024; Dörsam et al. 2022).

The present study focused on data from the validated self‐report questionnaires ED Examination Questionnaire (EDE‐Q) (Hilbert and Tuschen‐Caffier 2016) and the depression module of the Patient Health Questionnaire (PHQ‐9) (Löwe et al. 2001), which were completed online by the mothers during the third trimester of pregnancy (T1), 3 months (T2) and 10 months postpartum (T3) and by the fathers at T2 and T3. Additionally, the subscales Body Image, Marital relationship and Attitudes to pregnancy and the baby of the maternal adjustment and maternal attitudes questionnaire (MAMA) (Kumar, Robson, and Smith 1984) and the subscales Marital relationship and Attitudes towards pregnancy and baby of the paternal adjustment and paternal attitudes questionnaire (PAPA) (Pinto et al. 2017) applied at T2 and T3 were analyzed. More detailed information on the questionnaires can be found in Appendix S1. Maternal sociodemographic data were assessed with a self‐report questionnaire administered at study inclusion and information from the medical record of prenatal and natal care were extracted.

Ethical approval was obtained from the ethics committee of the medical faculty of the Eberhard‐Karls‐University and the University Hospital Tübingen (219/2018BO1). All study participants provided written informed consent.

Statistical analysis was performed using IBM SPSS Statistics for Windows, Version 28.0. Sample characteristics are presented as means and standard deviations or percentages. Intergroup differences were assessed with Fisher's exact test for nominal variables. For metric variables, *t*‐tests for normally distributed variables and Mann–Whitney *U*‐tests for not normally distributed variables were used. Normal distribution was checked by using histograms and Shapiro–Wilk test. Bonferroni‐Holm correction was used for multiple comparisons. Friedman tests with Dunn‐Bonferroni correction were used to investigate possible changes regarding EDE‐Q and PHQ‐9 scores for mothers over all three measurement points. Changes in the MAMA and PAPA scores as well as changes regarding paternal EDE‐Q and PHQ‐9 scores from T2 to T3 were tested with paired *t*‐tests or Wilcoxon tests. Effect sizes were quantified using either Phi‐coefficient *ϕ*, the correlation coefficient *r*, or Cohen's *d*. The classification of these effect sizes was as follows: small (*ϕ*, *r* = 0.10, *d* = 0.20), medium (*ϕ*, *r* = 0.30, *d* = 0.50), and large (*ϕ*, *r* = 0.50, *d* = 0.80) (Cohen 2016). Correlations between maternal and paternal characteristics were tested using Bravais‐Pearson correlation. The level of statistical significance was defined as *p* < 0.05.

## 3 Results

Fifty‐seven women and 56 partners were recruited in pregnancy. Twenty‐four women had an ED history (AN = 16, BN = 4, other specified feeding or ED (OSFED) = 3, binge‐ED (BED) = 1), 12 of which were in remission at study entry. Drop‐out was low, with one participant lost at T2 and a total of three participants reporting no data at T3. Sample characteristics can be found in Table 1 (see Appendix S2, S4 and S5 for further details).

**TABLE 1:** Parental characteristics.

	Total sample		ED		HC		ES	
	%	*n*	%	*n*	%	*n*	*p*	*φ*
Maternal characteristics (T1)								
Sample size	100	57	42	24	58	33	n.a.	
Nullipara	68.4	39	66.7	16	69.7	23	1.000^c^	0.032
Primipara	21.1	12	20.8	5	21.2	7		
Pluripara	10.6	6	12.5	3	9.1	3		
German nationality	93.0	53	100.0	24	87.9	29	0.130^c^	0.234
University degree	73.7	42	66.7	16	78.8	26	0.368^c^	−0.136
In a relationship	96.5	55	91.7	22	100.0	33	0.173^c^	−0.224
Family income ≥ 4000 €	56.1	32	50.0	12	60.6	20	0.589^c^	−0.106
Mental health comorbidities	10.5	6	20.8	5	3.0	1	0.073^c^	−0.286
Self‐reported physical complaints during pregnancy	77.2	44	91.7	22	66.7	22	0.030* ^c^	−0.294
Conspicuous course of birth^d^	22.8	13	16.7	4	27.3	9	0.524^c^	0.125
	M ± SD	*n*	M ± SD	*n*	M ± SD	*n*	*p*	*d*/*r*
Age (years)	31.30 ± 4.18	57	31.83 ± 3.99	24	30.91 ± 4.33	33	0.415^a^	0.221
BMI (kg/m^2^)	25.42 ± 4.15	57	25.78 ± 5.64	24	25.16 ± 2.66	33	0.734^b^	0.045
Gestational week	30.96 ± 2.81	57	30.79 ± 3.05	24	31.09 ± 2.66	33	0.442^b^	0.102
Gestational weight gain (kg)	12.76 ± 4.86	52	11.19 ± 5.45	21	13.82 ± 4.19	31	0.055^a^	0.556
Paternal characteristics (T2)								
Sample size	100	56	41	23	59	33		
Age (years)	35.23 ± 7.13	52	34.91 ± 6.35	23	35.48 ± 7.80	29	0.956^b^	0.008
BMI (kg/m^2^)	26.28 ± 4.11	54	27.52 ± 5.64	22	25.42 ± 2.34	32	0.291^b^	0.144
Paternal leave (weeks)	6.85 ± 6.38	55	6.52 ± 5.15	23	7.09 ± 7.21	32	0.666^b^	0.058

*Note*: Data presented as mean (M) ± standard deviation (SD) or %.

Abbreviations: BMI, body mass index; ED, eating disorder group; ES, effect size; HC, healthy control group.

^a^ Student's *t*‐test.

^b^ Mann‐Whitney‐*U*‐test.

^c^ Fisher's exact test.

^d^ Participant's self‐report.

* *p* <.05.

### 3.1 Eating Disorder Psychopathology

**FIGURE 1:**
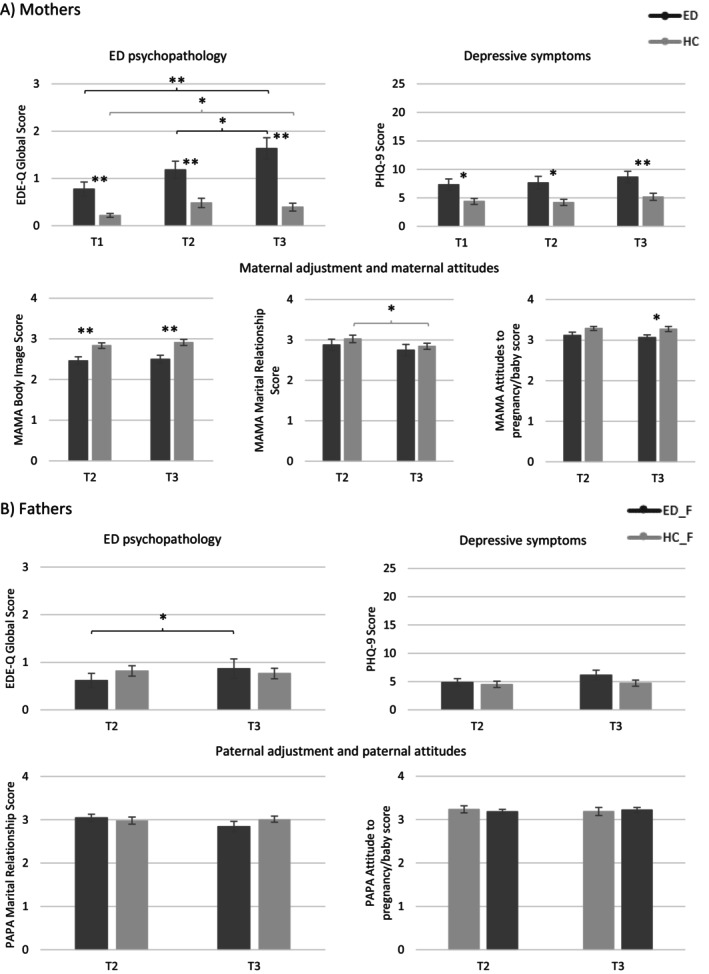
Course of parental eating pathology, depressive symptoms and adjustment to parenthood. ED, eating disorder; ED_F, partners of women with eating disorder history; EDE_Q, eating disorder examination‐questionnaire; HC, healthy control; HC_F, partners of women without eating disorder history; MAMA, maternal adjustment and maternal attitudes questionnaire; PAPA, paternal adjustment and paternal attitudes questionnaire; PHQ‐9, patient health questionnaire‐9.*p < 0.05; **p < 0.01.

Across all measurement points, women with ED history scored significantly higher on the EDE‐Q global score than women without ED history (T1: *Z* = −3.470, *p* < 0.001, *r* = 0.46; T2: *Z* = −3.654, *p* < 0.001, *r* = 0.48; T3: *Z* = −4.490, *p* < 0.001, *r* = 0.60, Figure 1). Regarding intragroup differences, global ED psychopathology increased significantly in both groups from T1 to T3 (ED: *Z* = −1.217, *p* < 0.001, *r* = 0.25; HC: *Z* = −0.697, *p* = 0.014, *r* = 0.12). This increase was larger in women with ED history (*Z* = −3.515, *p* < 0.001, *r* = 0.47). For women in the ED group, a significant increase in global ED psychopathology was also observed from T2 to T3 (*Z* = −0.739, *p* = 0.037, *r* = 0.15).

Partners showed no significant differences in EDE‐Q global scores at T2 and T3 (Figure 1). Partners of women with ED history reported significantly more ED psychopathology at T3 compared to T2 (*Z* = −1.981, *p* = 0.048, *r* = 0.42). Partners of women without ED history had no change in their ED psychopathology from T2 to T3.

### 3.2 Depressive Symptoms

Across all measurement points, mothers with ED history had significantly higher depression scores compared to women in the control group (T1: *Z* = −2.542, *p* = 0.011, *r* = 0.34; T2: *Z* = −2.354, *p* = 0.019, *r* = 0.31; T3: *Z* = −2.804, *p* = 0.005, *r* = 0.38; Figure 1). Depressive symptoms did not change significantly over time in both groups.

There were no significant differences between partners of women with and without ED history regarding PHQ‐9 scores across the two measurement points (Figure 1). Furthermore, there were no time effects in both groups.

### 3.3 Adjustment to Parenthood

Mothers in both groups did not significantly differ in their rating of the Marital Relationship (Figure 1). Mothers in the control group scored higher on the Body Image subscale on both measurement points (T2: *t*(55) = −3.292, *p* = 0.006, *d* = 0.88; T3: *Z* = −3.146, *p* = 0.006, *r* = 0.42) and on the attitudes towards pregnancy and baby subscale at T3 (*Z* = −2.811, *p* = 0.010, *r* = 0.38) compared to mothers with ED history. There was a significant decrease in the Marital Relationship subscale score for women in the HC group from T2 to T3 (*t*(32) = 2.537, *p* = 0.048, *d* = 0.44). No other time effects were found.

Fathers did not differ regarding the PAPA subscale scores (Figure 1). Further, no time effects were found in either group.

### 3.4 Correlations With ED Psychopathology

Maternal EDE‐Q global scores were not associated with paternal EDE‐Q global scores and paternal PHQ‐9 scores at T2. There was a significant negative correlation between the maternal EDE‐Q global scores and the PAPA subscale scores attitudes towards pregnancy and baby in the ED group with medium effect (*r* = −0.440, *p* = 0.036), while no significant correlations were found in the HC group between maternal EDE‐Q global scores and paternal adjustment scores (Appendix S3).

At T3, there were no significant correlations between maternal ED psychopathology and paternal characteristics, neither in the HC nor in the ED group.

No correlations between paternal eating behavior and maternal characteristics were found in the individual groups or across the time periods.

## 4 Discussion

This study longitudinally investigated ED psychopathology, depressive symptoms, and adjustment to parenthood in mothers with and without ED history and their partners during the first year after childbirth. Mothers with ED history had significantly more severe ED psychopathology and higher depression scores, as well as lower levels of adjustment to motherhood compared to the HC group across all measurement points. Further, ED psychopathology increased significantly from late pregnancy to 10 months postpartum in both groups. Hence, our findings mostly support previous research (Astrachan‐Fletcher et al. 2008; Baskin, Meyer, and Galligan 2021; Koubaa, Hällström, and Hirschberg 2008; Tan et al. 2023). The increase in ED psychopathology postpartum in both groups highlights the vulnerability of women in this period regarding disordered eating, especially in women with ED history, however, effect sizes were small. The medium and strong effect regarding intergroup differences in the MAMA subscale Body Image reflect the stronger struggles with body‐related aspects in women with ED history. We further confirmed the findings of Koubaa, Hällström, and Hirschberg (2008) that women with ED history have less positive attitudes towards pregnancy and the baby compared to HC mothers. The marital relationships of women without ED appear to be adversely affected during the initial postpartum year, with a medium effect. However, mean subscale scores remained higher than those observed in the ED group. We found no increases in depressive symptomatology after childbirth in neither group. This may be due to the overall low severity of ED psychopathology in our sample and may be different in a study population with more severely ill patients. Overall, women who have not previously experienced an ED appear to demonstrate greater resilience in responding to novel challenges they encounter.

The findings in relation to the fathers are not in line with those of previous research (Batchelor et al. 2022; Linville et al. 2016; O'Connor, Daly, and Higgins 2019; Schmit and Bell 2017). The lack of differences in ED psychopathology between the groups in our study may be explained by the overall relatively low levels of ED symptoms. However, ED psychopathology of fathers in the ED group significantly increased from T2 to T3, and paternal adjustment was significantly correlated with maternal ED psychopathology three months after birth, with medium effects. Hence, findings on paternal mental health are mixed: Fathers whose partners report higher ED severity tended to struggle with their role adjustment after childbirth, reflecting a specific burden in families affected by ED (history). On the other hand, paternal self‐report on low ED symptoms and depression might be seen as encouraging. Furthermore, fathers may play a protective role in families with maternal ED history by serving as a role model for healthy eating behavior for their children (Savage, Fisher, and Birch 2007).

### 4.1 Strengths and Limitations

This is the first longitudinal study on mental health outcomes in mothers with ED history and their partners in early and later postpartum stages. The participants of this study were recruited from the general population and group allocation was based on the EDE. The low drop‐out rate prohibits data loss and attrition bias.

Limitations include the small sample size, the representation of mainly women diagnosed with AN in the ED group, and the use of non‐validated questionnaires to assess parental adjustment. Further, paternal outcomes were assessed starting postpartum, so, as opposed to mothers, we cannot compare to pre‐birth values in fathers. Lastly, some recruitment took place during the COVID‐19 pandemic, which may have influenced the results.

## 5 Conclusions and Implications

Mothers with an ED history report higher levels of psychological distress and adjustment difficulties in the first year after childbirth compared to mothers without ED history. Maternal ED severity correlated with parental adjustment; however, this does not necessarily manifest in psychological distress. These results highlight the need for a close monitoring and consistent care of women affected by ED during the vulnerable postpartum period and also highlight adjustment needs of partners of severely affected women. Further qualitative approaches would help to deepen the knowledge of the paternal experiences during this period. Of note, partners of women with ED may play a supportive role in the family system affected by ED. Future research should consider the investigation of the impact on paternal mental health on the offspring in families with ED history.

## Author Contributions

**Jana Katharina Throm:** formal analysis, investigation, project administration, visualization, writing – original draft. **Annica Franziska Dörsam:** conceptualization, investigation, methodology, project administration, writing – review and editing. **Nadia Micali:** conceptualization, funding acquisition, methodology, writing – review and editing. **Hubert Preissl:** conceptualization, funding acquisition, writing – review and editing. **Katrin Elisabeth Giel:** conceptualization, funding acquisition, methodology, project administration, supervision, writing – review and editing.

## Conflicts of Interest

The authors declare no conflicts of interest.

## Supporting information


Appendix S1.

Appendix S2.

Appendix S3.

Appendix S4.

Appendix S5.

## Data Availability

The data that support the findings of this study are available from the corresponding author upon reasonable request.
