# Supplementary material for: Dyadic Psychopathology and Adjustment to Parenthood in Families With and Without Eating Disorder History—Findings From a Longitudinal Study
Source: Int J Eat Disord. 2024 Nov 27;58(2):452–8. doi: 10.1002/eat.24338 (PMC11861874; doi:10.1002/eat.24338)
Supplement: Supplementary file 1 — Appendix S1. [file EAT-58-452-s003.docx]

Supplementary Material 1 – Psychometric measures

*Eating Disorder Examination-Questionnaire.* Severity of ED behavior was assessed with the German Version of the Eating Disorder Examination-Questionnaire (EDE-Q) (Hilbert & Tuschen-Caffier, 2016). 22 items divided in four subscales (*Restraint, Eating Concern, Weight Concern,* *Shape Concern)* ascertain the intensity of ED psychopathology of the last 28 days, with six items recording diagnostically relevant behavior. Frequencies and intensity of ED-specific characteristics were rated on 7-point Likert-scales, whereby higher scores represented more severe ED psychopathology. The EDE-Q global score showed high internal consistency (α = .86) for mothers and acceptable internal consistency (α = .72) for fathers in our study at baseline.

*Patient Health Questionnaire-9.* The depression module of the Patient Health Questionnaire (PHQ-D) was used for the assessment of depressive symptomatology (Löwe, 2001). The 9 items were scored on a 4-point Likert-scale, with a score lower than 5 indicating the absence of a depressive disorder (Löwe, 2001). The PHQ-D had high internal consistency (α = .84) for mothers and acceptable internal consistency (α = .74) for fathers in our study at baseline.

*Maternal Adjustment and Maternal Attitudes Questionnaire (MAMA).* The Maternal Adjustment and Maternal Attitudes Questionnaire (MAMA) is a self-report questionnaire for the measurement of a mother’s perceptions of her body image, the marital relationship and attitudes towards pregnancy and the baby (Kumar et al., 1984). The questionnaire was translated from English into German by a member of the study team. Subsequently, this version was then translated back into the source language by a native speaker and compared with the original version. In the present study, we used the MAMA subscales *Body Image, Marital relationship* and *Attitudes to pregnancy and the baby.* The adjusted questionnaire comprised 36 items, rated on a 4-point Likert-scale. Higher scores indicated higher levels of adjustment and adequate attitudes towards motherhood. The MAMA showed high internal consistency (α = .87) in our study sample at baseline.

*Paternal Adjustment and Paternal Attitudes Questionnaire (PAPA).* Paternal adjustment and paternal attitudes in the first year after childbirth was assessed with a shortened version of the Paternal Adjustment and Paternal Attitudes Questionnaire (PAPA) (Pinto et al., 2017). The translation was completed similarly to the MAMA questionnaire. The two subscales *Marital relationship,* *Attitudes to pregnancy and the baby* with ten items respectively, were applied. The items were scored on 4-point Likert-scales, with higher scores indicating higher levels of adjustment and adequate attitudes. Internal consistency was acceptable in our study (α = .75) at baseline.
