# Supplementary material for: Dyadic Psychopathology and Adjustment to Parenthood in Families With and Without Eating Disorder History—Findings From a Longitudinal Study
Source: Int J Eat Disord. 2024 Nov 27;58(2):452–8. doi: 10.1002/eat.24338 (PMC11861874; doi:10.1002/eat.24338)
Supplement: Supplementary file 2 — Appendix S2. [file EAT-58-452-s002.docx]

Supplementary Material 2: Inter- and intragroup differences

| *1. Mothers* |  |  |  |  |  |  |  |  |  |  |  |  |  |  |  |  |  |  |
| --- | --- | --- | --- | --- | --- | --- | --- | --- | --- | --- | --- | --- | --- | --- | --- | --- | --- | --- |
| a) Group differences | |  |  |  |  |  |  |  |  |  |  |  |  |  |  |  |  |  |
|  | **T1** |  |  |  |  |  | **T2** |  |  |  |  |  | **T3** |  |  |  |  |  |
|  | ED |  | HC |  |  |  | ED |  | HC |  |  |  | ED |  | HC |  |  |  |
|  | *M ± SD* | *n* | *M ± SD* | *n* | *p* | *ES* | *M ± SD* | *n* | *M ± SD* | *n* | *p* | *ES* | *M ± SD* | *n* | *M ± SD* | *n* | *p* | *ES* |
| EDE-Q | 0.78 ± 0.72 | 24 | 0.22 ± 0.23 | 33 | **.001^b^** | .46 | 1.18 ± 0.89 | 24 | 0.48 ± 0.58 | 33 | **< .001^b^** | .48 | 1.63 ± 1.09 | 23 | 0.39 ± .047 | 33 | **< .001^b^** | .60 |
| PHQ | 7.33 ± 4.90 | 24 | 4.39 ± 2.96 | 33 | **.011^b^** | .34 | 7.67 ± 5.56 | 24 | 4.21 ± 3.05 | 33 | **.019^b^** | .31 | 8.65 ± 4.84 | 23 | 5.18 ± 3.60 | 33 | **.005^b^** | .38 |
| MAMA BI | n.a. | n.a. | n.a. | n.a. | n.a. | n.a. | 2.46 ± 0.47 | 24 | 2.84 ± 0.39 | 33 | **.006^a^** | .88 | 2.50 ± 0.46 | 23 | 2.91 ± 0.42 | 33 | **.006^b^** | .42 |
| MAMA MR | n.a. | n.a. | n.a. | n.a. | n.a. | n.a. | 2.88 ± 0.69 | 24 | 3.03 ± 0.53 | 33 | .588^b^ | .07 | 2.75 ± 0.66 | 23 | 2.84 ± 0.44 | 33 | .557^a^ | .17 |
| MAMA APC | n.a. | n.a. | n.a. | n.a. | n.a. | n.a. | 3.12 ± 0.39 | 24 | 3.29 ± 0.30 | 33 | .196^b^ | .22 | 3.07 ± 0.31 | 23 | 3.27 ± 0.36 | 33 | **.010^b^** | .38 |
|  |  |  |  |  |  |  |  |  |  |  |  |  |  |  |  |  |  |  |
| b) Time differences | |  |  |  |  |  |  |  |  |  |  |  |  |  |  |  |  |  |
|  | **T1 vs. T2** |  |  |  |  |  | **T2 vs. T3** |  |  |  |  |  | **T1 vs. T3** |  |  |  |  |  |
|  | ED |  | HC |  |  |  | ED |  | HC |  |  |  | ED |  | HC |  |  |  |
|  | *p* | *ES* | *p* | *ES* |  |  | *p* | *ES* | *p* | *ES* |  |  | *p* | *ES* | *p* | *ES* |  |  |
| EDE-Q | .314^c^ | .10 | .058^c^ | .10 |  |  | .**037^c^** | .15 | 1.000^c^ | .02 |  |  | **< .001^c^** | .25 | **.014^c^** | .12 |  |  |
| PHQ | .871^d^ | .03 | .487^d^ | .12 |  |  | .151^d^ | .30 | .058^d^ | .33 |  |  | .116^d^ | .33 | .152^d^ | .25 |  |  |
| MAMA BI | n.a. | n.a. | n.a. | n.a. |  |  | .497^a^ | .14 | .230^a^ | .28 |  |  | n.a. | n.a. | n.a. | n.a. |  |  |
| MAMA MR | n.a. | n.a. | n.a. | n.a. |  |  | .716^d^ | .19 | **.048^a^** | .44 |  |  | n.a. | n.a. | n.a. | n.a. |  |  |
| MAMA APC | n.a. | n.a. | n.a. | n.a. |  |  | .990^a^ | .21 | .991^d^ | .00 |  |  | n.a. | n.a. | n.a. | n.a. |  |  |
|  |  |  |  |  |  |  |  |  |  |  |  |  |  |  |  |  |  |  |
| *2. Fathers* |  |  |  |  |  |  |  |  |  |  |  |  |  |  |  |  |  |  |
| a) Group differences | |  |  |  |  |  |  |  |  |  |  |  |  |  |  |  |  |  |
|  | **T1** |  |  |  |  |  | **T2** |  |  |  |  |  | **T3** |  |  |  |  |  |
|  | ED |  | HC |  |  |  | ED |  | HC |  |  |  | ED |  | HC |  |  |  |
|  | *M ± SD* | *n* | *M ± SD* | *n* | *p* | *ES* | *M ± SD* | *n* | *M ± SD* | *n* | *p* | *ES* | *M ± SD* | *n* | *M ± SD* | *n* | *p* | *ES* |
| EDE-Q | n.a. | n.a. | n.a. | n.a. | n.a. | n.a. | 0.62 ± 0.72 | 23 | 0.82 ± 0.61 | 32 | .069^b^ | .25 | 0.87 ± 0.96 | 22 | 0.76 ± 0.62 | 32 | .778^b^ | .04 |
| PHQ | n.a. | n.a. | n.a. | n.a. | n.a. | n.a. | 4.87 ± 3.11 | 23 | 4.50 ± 3.14 | 32 | .491^b^ | .09 | 6.14 ± 4.13 | 22 | 4.72 ± 3.05 | 32 | .214^b^ | .17 |
| PAPA MR | n.a. | n.a. | n.a. | n.a. | n.a. | n.a. | 3.04 ± 0.42 | 23 | 2.98 ± 0.46 | 32 | .610^a^ | .14 | 2.84 ± 0.57 | 22 | 3.01 ± 0.40 | 32 | .626^b^ | .14 |
| PAPA APC | n.a. | n.a. | n.a. | n.a. | n.a. | n.a. | 3.23 ± 0.40 | 23 | 3.18 ± 0.28 | 32 | .404^b^ | .17 | 3.19 ± 0.44 | 22 | 3.22 ± 0.32 | 32 | .804^b^ | .03 |
|  |  |  |  |  |  |  |  |  |  |  |  |  |  |  |  |  |  |  |
| b) Time differences | |  |  |  |  |  |  |  |  |  |  |  |  |  |  |  |  |  |
|  | **T1 vs. T2** |  |  |  |  |  | **T2 vs. T3** |  |  |  |  |  | **T1 vs. T3** |  |  |  |  |  |
|  | ED |  | HC |  |  |  | ED |  | HC |  |  |  | ED |  | HC |  |  |  |
|  | *p* | *ES* | *p* | *ES* |  |  | *p* | *ES* | *p* | *ES* |  |  | *p* | *ES* | *p* | *ES* |  |  |
| EDE-Q | n.a. | n.a. | n.a. | n.a. |  |  | **.048^d^** | .42 | .418^a^ | .15 |  |  | n.a. | n.a. | n.a. | n.a. |  |  |
| PHQ | n.a. | n.a. | n.a. | n.a. |  |  | .084^d^ | .37 | .617^a^ | .09 |  |  | n.a. | n.a. | n.a. | n.a. |  |  |
| PAPA MR | n.a. | n.a. | n.a. | n.a. |  |  | .437^a^ | .17 | .880^a^ | .13 |  |  | n.a. | n.a. | n.a. | n.a. |  |  |
| PAPA APC | n.a. | n.a. | n.a. | n.a. |  |  | .062^d^ | .46 | .485^d^ | .12 |  |  | n.a. | n.a. | n.a. | n.a. |  |  |

*Abbreviations: eating disorder (ED), healthy control (HC), Eating Disorder Examination-Questionnaire (EDE-Q), Patient Health Questionnaire-9 (PHQ-9), Maternal Adjustment and Maternal Attitudes Questionnaire (MAMA), Paternal Adjustment and Paternal Attitudes Questionnaire (PAPA), Body Image (BI), Marital Relationship (MR), Attitudes towards pregnancy and child (APC), ^a^Student’s t-Test, ^b^Mann-Whitney-U-Test, ^c^Friedman-Test, ^d^Wilcoxon-Test*
