# Supplementary material for: Dyadic Psychopathology and Adjustment to Parenthood in Families With and Without Eating Disorder History—Findings From a Longitudinal Study
Source: Int J Eat Disord. 2024 Nov 27;58(2):452–8. doi: 10.1002/eat.24338 (PMC11861874; doi:10.1002/eat.24338)
Supplement: Supplementary file 3 — Appendix S3. [file EAT-58-452-s001.docx]

Supplementary Material 3: Correlation analysis

| ***1. Three months after birth*** | | |  |  |  |  |  |  |  |  |  |
| --- | --- | --- | --- | --- | --- | --- | --- | --- | --- | --- | --- |
|  |  | *a) ED group* |  |  |  |  | *b) HC group* |  |  |  |  |
|  |  | EDE-Q Father | PHQ9 Father | PAPA MR | PAPA APC |  | EDE-Q Father | PHQ9 Father | PAPA MR | PAPA APC |  |
| EDE-Q Mother | *r* | -.067 | .390 | -.263 | -.440 |  | .169 | .065 | -.024 | .077 |  |
|  | *p* | .760 | .066 | .225 | **.036*** |  | .355 | .726 | .898 | .674 |  |
|  | *n* | 23 | 23 | 23 | 23 |  | 32 | 32 | 32 | 32 |  |
|  |  |  |  |  |  |  |  |  |  |  |  |
|  |  | EDE-Q Mother | PHQ9 Mother | MAMA MR | MAMA APC | MAMA BI | EDE-Q Mother | PHQ9 Mother | MAMA MR | MAMA APC | MAMA BI |
| EDE-Q Father | *r* | -.067 | -.233 | -.321 | .302 | .189 | .169 | -048 | .142 | .082 | .031 |
|  | *p* | .760 | .284 | .135 | .162 | .388 | .355 | ,793 | .437 | .657 | .867 |
|  | *n* | 23 | 23 | 23 | 23 | 23 | 32 | 32 | 32 | 32 | 32 |
|  |  |  |  |  |  |  |  |  |  |  |  |
|  |  |  |  |  |  |  |  |  |  |  |  |
| ***2. Ten months after birth*** | | |  |  |  |  |  |  |  |  |  |
|  |  | *a) ED group* |  |  |  |  | *b) HC group* |  |  |  |  |
|  |  | EDE-Q Father | PHQ9 Father | PAPA MR | PAPA APC |  | EDE-Q Father | PHQ9 Father | PAPA MR | PAPA APC |  |
| EDE-Q Mother | *r* | -.087 | .280 | -.088 | -.212 |  | -.105 | -.189 | .099 | .081 |  |
|  | *p* | .699 | .208 | .697 | .343 |  | .569 | .301 | .589 | .660 |  |
|  | *n* | 22 | 22 | 22 | 22 |  | 32 | 32 | 32 | 32 |  |
|  |  |  |  |  |  |  |  |  |  |  |  |
|  |  | EDE-Q Mother | PHQ9 Mother | MAMA MR | MAMA APC | MAMA BI | EDE-Q Mother | PHQ9 Mother | MAMA MR | MAMA APC | MAMA BI |
| EDE-Q Father | *r* | -.087 | .010 | -.108 | .037 | -.126 | -.105 | -.047 | -.098 | .125 | .042 |
|  | *p* | .699 | .964 | .633 | .871 | .575 | .569 | .797 | .595 | .495 | .819 |
|  | *n* | 22 | 22 | 22 | 22 | 22 | 32 | 32 | 32 | 32 | 32 |

*Abbreviations: eating disorder (ED), healthy control (HC), Eating Disorder Examination-Questionnaire (EDE-Q), Patient Health Questionnaire-9 (PHQ-9), Maternal Adjustment and Maternal Attitudes Questionnaire (MAMA), Paternal Adjustment and Paternal Attitudes Questionnaire (PAPA), Marital Relationship (MR), Attitudes towards pregnancy and child (APC), Body image (BI), r (Pearson correlation coefficient*

** = p < .05*
