# Supplementary material for: Dyadic Psychopathology and Adjustment to Parenthood in Families With and Without Eating Disorder History—Findings From a Longitudinal Study
Source: Int J Eat Disord. 2024 Nov 27;58(2):452–8. doi: 10.1002/eat.24338 (PMC11861874; doi:10.1002/eat.24338)
Supplement: Supplementary file 4 — Appendix S4. [file EAT-58-452-s004.docx]

Supplementary Material 4: Maternal EDE-Q diagnostic items

|  | **T1** |  |  |  |  |  | **T2** |  |  |  |  |  | **T3** |  |  |  |  |  |
| --- | --- | --- | --- | --- | --- | --- | --- | --- | --- | --- | --- | --- | --- | --- | --- | --- | --- | --- |
|  | **ED** |  | **HC** |  |  |  | **ED** |  | **HC** |  |  |  | **ED** |  | **HC** |  |  |  |
|  | *M ± SD* | *n* | *M ± SD* | *n* | *p* | *ES* | *M ± SD* | *n* | *M ± SD* | *n* | *p* | *ES* | *M ± SD* | *n* | *M ± SD* | *n* | *p* | *ES* |
| **EDE-Q diagnostic items** |  |  |  |  |  |  |  |  |  |  |  |  |  |  |  |  |  |  |
| Objective overeating | 2.83 ± 6.54 | 24 | 1.15 ± 2.20 | 33 | .780 | .04 | 3.29 ± 7.28 | 24 | 2.52 ± 5.27 | 33 | .582 | .08 | 2.87 ± 6.62 | 23 | 1.55 ± 2.88 | 33 | .694 | .05 |
| Loss of control over eating | 1.04 ± 2.82 | 24 | 0.18 ± 0.53 | 33 | .314 | .13 | 2.46 ± 5.04 | 24 | 0.18 ± 0.73 | 33 | .013 | .33 | 1.17 ± 2.48 | 23 | 0.45 ± 2.11 | 33 | .083 | .23 |
| Objective binge-eating | 0.92 ± 2.55 | 24 | 0.27 ± 1.07 | 33 | .327 | .13 | 1.83 ± 4.25 | 24 | 0.12 ± 0.55 | 33 | .006 | .36 | 0.96 ± 2.35 | 23 | 0.15 ± 0.87 | 33 | .030 | .29 |
| Self-induced vomiting | 1.58 ± 5.99 | 24 | 0.00 ± 0.00 | 33 | .094 | .22 | 2.21 ± 6.89 | 24 | 0.00 ± 0.00 | 33 | .039 | .27 | 1.57 ± 6.37 | 23 | 0.00 ± 0.00 | 33 | .035 | .28 |
| Abuse of laxatives | 0.00 ± 0.00 | 24 | 0.00 ± 0.00 | 33 | 1.000 | .00 | 0.00 ± 0.00 | 24 | 0.00 ± 0.00 | 33 | 1.000 | .00 | 0.00 ± 0.00 | 23 | 0.00 ± 0.00 | 33 | 1.000 | .00 |
| Driven physical exercise | 1.33 ± 4.27 | 24 | 0.00 ± 0.00 | 33 | .016 | .32 | 1.29 ± 4.19 | 24 | 0.12 ± 0.49 | 33 | .163 | .18 | 0.61 ± 1.37 | 23 | 0.00 ± 0.00 | 33 | .005 | .37 |

*Abbreviations: ED (eating disorder group), HC (healthy control group)*
