# Supplementary material for: Dyadic Psychopathology and Adjustment to Parenthood in Families With and Without Eating Disorder History—Findings From a Longitudinal Study
Source: Int J Eat Disord. 2024 Nov 27;58(2):452–8. doi: 10.1002/eat.24338 (PMC11861874; doi:10.1002/eat.24338)
Supplement: Supplementary file 5 — Appendix S5. [file EAT-58-452-s005.docx]

Supplementary Matrial 5: Subgroup analysis

1. *EDE-Q: There was no statistically significant interaction between time and group (Greenhouse–Geisser F (1.868, 39.238) = .258, p = .759, partial η² = .01, Huynh-Feldt: F (2.000, 42.000) = .258, p = .774, partial η² = .01). However, there was a significant main effect for group (F (1,21) = 12.644, p = .002, partial η² = .376, d = 1.476) and time (Greenhouse-Geisser F (1.868, 39.238) = 10.545, p < .001, partial η² = .33; Huynh-Feldt: F (2.000, 42.000) = 10.545, p < .001, partial η² = .33). Pairwise comparison showed a significant difference only between T1 and T3 (p = .002, d = .868, corrected d = .946), with higher scores at the latter point.*
2. *PHQ: There was no statistically significant interaction between time and group (Greenhouse–Geisser:* F *(1.914, 40.198) = .044,* p*= .952, partial η² = .00; Huynh-Feldt: F (2.000, 42.000) = .044, p = .957, partial η² = .00). Furthermore, there was no significant change over time (Greenhouse-Geisser: F (1.914, 40.198) = 1.210, p = .307, partial n² = .054; Huynh-Feldt: F(2.000, 42.000) = 1.210, p = .308, partial n² = .054). However, we found a significant main effect for group (F (1, 21) = 5.017, p = .036, partial n²= .19, d = .901), with higher scores in the group with active ED symptoms.*
